# Vitamin D and COVID-19: How Much Vitamin D Does a Man Need?

**DOI:** 10.3390/nu13124311

**Published:** 2021-11-29

**Authors:** Igor N. Sergeev

**Affiliations:** Department of Health and Nutritional Sciences, South Dakota State University, Brookings, SD 57007, USA; igor.sergeev@sdstate.edu

A number of observational studies and reviews on the potential role of vitamin D in COVID-19 have been published since the beginning of this ongoing global pandemic. In the observational studies, “low” vitamin D intake and “deficient” vitamin D status have been often linked to the increased risk, severity and duration of COVID-19, with implications or recommendations for using high doses of vitamin D in the prophylaxis or even treatment of this disease (e.g., [1]). Those recommendations are in the context of efforts aimed at promoting high intake of vitamin D for the prevention and treatment of numerous diseases and maintaining good health. I argued in a recently published review [2] that recommending the intake of vitamin D in amounts exceeding those established by the U.S. Institute of Medicine (IOM) [3] to maintain concentration of the transport form of vitamin D, 25-hydroxivitamin D (25(OH)D), above the sufficient level of 20–30 ng/mL (also established by this organization) are not based on mechanistic evidence. I emphasized that “…it is critical to consider that the normal, physiological concentration of the active, hormonal form of vitamin D_3_—1,25(OH)_2_D_3_—in the blood and target tissues of healthy adults is regulated and maintained within a broad range of concentrations of its precursor, 25(OH)D_3_” [2]. 25(OH)D per se has no vitamin D biological or hormonal activity at “normal” concentrations but demonstrates chronic (50–200 ng/mL) and acute (200–1000 ng/mL) vitamin D toxicity at high concentrations (due to its non-selective binding to the vitamin D receptor) [2,4,5,6].

The main practical conclusion based on the mechanistic understanding of vitamin D_3_‘s role as the precursor of the hormone 1,25-dihydroxyvitamin D_3_ (1,25(OH)_2_D_3_) is that physiological concentration of this hormone is homeostatically and precisely controlled and that it can be maintained even at “low” and “deficient” concentrations of the substrate, 25(OH)D_3_, for a long period of time (up to several months) [2,4]. Because production of 25(OH)D_3_ is not regulated in a homeostatic fashion, a high and sustained intake of vitamin D_3_ (>4000 IU/d) will increase the concentration of 25(OH)D_3_ to >50 ng/mL, thus markedly elevating the risk of chronic vitamin D toxicity. Furthermore, “…in the enzymatic reaction producing 1,25(OH)_2_D_3_, the substrate, 25(OH)D_3_, mechanistically functions not as a rate-limiting, but rather as an inhibitory substrate (i.e., high concentrations of 25(OH)D_3_ in the blood will inhibit production of 1,25(OH)_2_D_3_ in kidneys)” [2], and this limits the value for the organism to maintain a high concentration of 25(OH)D_3_ but safeguards against vitamin D toxicity due to 1,25(OH)_2_D_3_.

Vitamin D is not a panacea but a rather toxic compound [3,4], which should be used in accordance with the principle *“primum non nocere”*. Vitamin D status is considered as “an excellent marker of general health status” [6], implying that significance of the observational and statistical evidence linking vitamin D status to disease is limited in the absence of demonstrated mechanisms. Discovery of the effective and specific therapeutic modalities for COVID-19 should be based on the understanding of mechanisms of infection and immune response, and it should not be distracted by the hope for finding treatment and preventive options, which are not supported by mechanistic evidence. A successful evidence-based health policy for COVID-19 should also rely on mechanistic information causally connecting the policy intervention (e.g., a high-dose vitamin D supplementation) to its outcomes.

The question in the title of this commentary mimics Leo Tolstoy’s short story title “How Much Land Does a Man Need?” In the quest to attain more and more land, a man was promised as much of it as he can encircle walking for a day, and he dies running and trying. This author’s answer to this question is: “Six feet from his head to his heels was all he needed”.
